# Direct and trans-generational effects of male and female gut microbiota in *Drosophila melanogaster*

**DOI:** 10.1098/rsbl.2016.0966

**Published:** 2017-07-19

**Authors:** Juliano Morimoto, Stephen J. Simpson, Fleur Ponton

**Affiliations:** 1 Department of Zoology, Edward Grey Institute, University of Oxford, South Parks Road, Oxford OX1 3PS, United Kingdom; 2 Charles Perkins Centre, University of Sydney, Camperdown, New South Wales 2006, Australia; 3 Programa de Pós-Graduação em Ecologia e Conservação, Federal University of Paraná, Curitiba 19031, CEP 81531-990, Brazil; 4 School of Life and Environmental Sciences, University of Sydney, Sydney 2050, Australia; 5 Department of Biological Sciences, Macquarie University, North Ryde, New South Wales 2109, Australia

**Keywords:** sexual conflict, mate choice, holobiont, polyandry, microbiome

## Abstract

There is increasing evidence of the far-reaching effects of gut bacteria on physiological and behavioural traits, yet the fitness-related consequences of changes in the gut bacteria composition of sexually interacting individuals remain unknown. To address this question, we manipulated the gut microbiota of fruit flies, *Drosophila melanogaster*, by monoinfecting flies with either *Acetobacter pomorum* (*AP*) or *Lactobacillus plantarum* (*LP*)*.* Re-inoculated individuals were paired in all treatment combinations. *LP-*infected males had longer mating duration and induced higher short-term offspring production in females compared with *AP*-infected males. Furthermore, females of either re-inoculation state mated with *AP-*infected males were more likely to have zero offspring after mating, suggesting a negative effect of *AP* on male fertility*.* Finally, we found that the effects of male and female gut bacteria interacted to modulate their daughters', but not sons' body mass, revealing a new trans-generational effect of parental gut microbiota. In conclusion, this study shows direct and trans-generational effects of the gut microbiota on mating and reproduction.

## 1. Introduction

In nature, virtually all surfaces and cavities of an animal's body are inhabited by microorganisms, many of which are in some way linked to the animal's physiology and behaviour. Among these microorganisms, one group is particularly functionally important: the bacteria inhabiting the gut [1]. In insects, gut bacteria have been shown to modulate developmental rate [2,3], metabolism [4,5] and nutritional physiology [6], as well as social and sexual interactions [7,8]. For instance, previous findings suggest that fruit flies, *Drosophila melanogaster*, prefer to mate with partners with gut bacteria composition similar to rather than different from their own—an example of positive assortative, homotypic mating [8]. If this choice evolved through evolutionary processes (e.g. sexual selection) or is subjected to rapid plastic responses, it is expected to confer reproductive benefits either directly (i.e. increased number of offspring) or indirectly (i.e. increased quality of the offspring), or both. Conversely, if changes in gut bacteria do not affect choice or affect mate choice randomly, then changes in gut bacteria composition should present no obvious reproductive nor behavioural associations. Unfortunately, most studies on gut bacteria effects have largely overlooked short- and long-term fitness-related traits (but see [9,10]). As a result, the reproductive consequences of the gut bacteria-induced mate choice remain elusive, and we still do not know the evolutionary influence of changes in gut bacteria.

In this study, we addressed this question by testing how manipulations of the gut bacteria of male and female *D. melanogaster* affected aspects of reproduction and offspring body mass. Recent studies show that, although the presence and abundance of particular species of gut bacteria in wild populations can be inconsistent [11], two species greatly influence the physiology and behaviour of *D. melanogaster*–*Acetobacter pomorum* (*AP*) and *Lactobacillus plantarum* (*LP*) [12]*.* For instance, both *AP* and *LP* benefit flies under nutritional stress [2,3] and modulate flies' amino acid appetite [10]. Given these effects, there was no *a priori* evidence to formulate predictions on the strain-specific effects of bacteria on flies' sexual behaviour and reproduction. However, based on a previous study by Sharon *et al*. [8] in *D. melanogaster*, we predicted shorter latency of females to mate, longer mating duration, higher offspring production and heavier offspring in homotypic mating treatments relative to heterotypic mating treatments if gut microbiota-induced assortative mating responds plastically or has evolved under sexual and natural selection.

## 2. Results and Discussion

**Figure 1.**
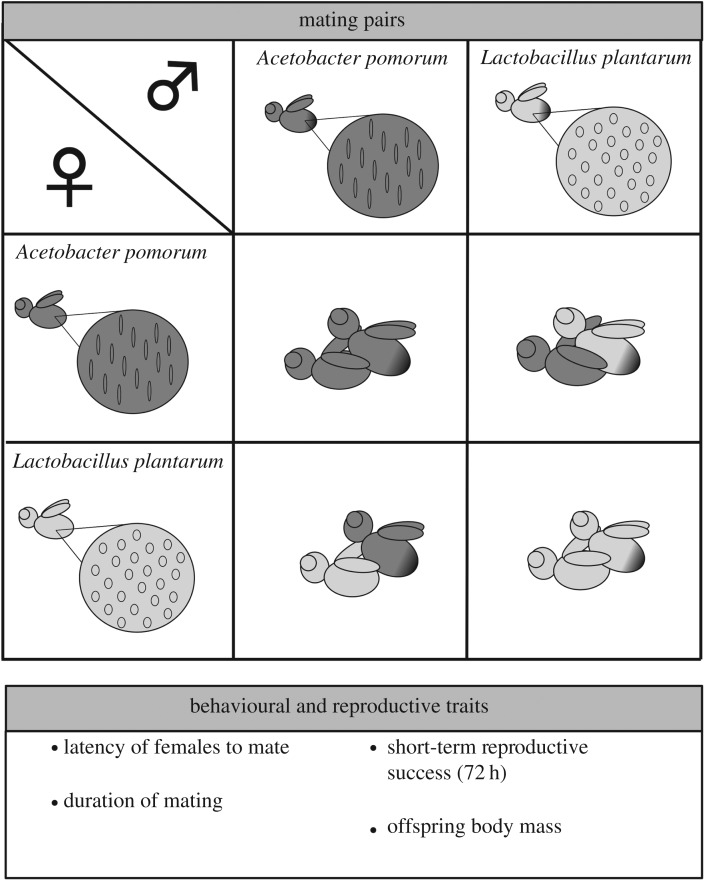
Schematic of the experimental design. Circles represent the gut of flies, each re-inoculated with either *AP* or *LP.*

**Figure 2.**
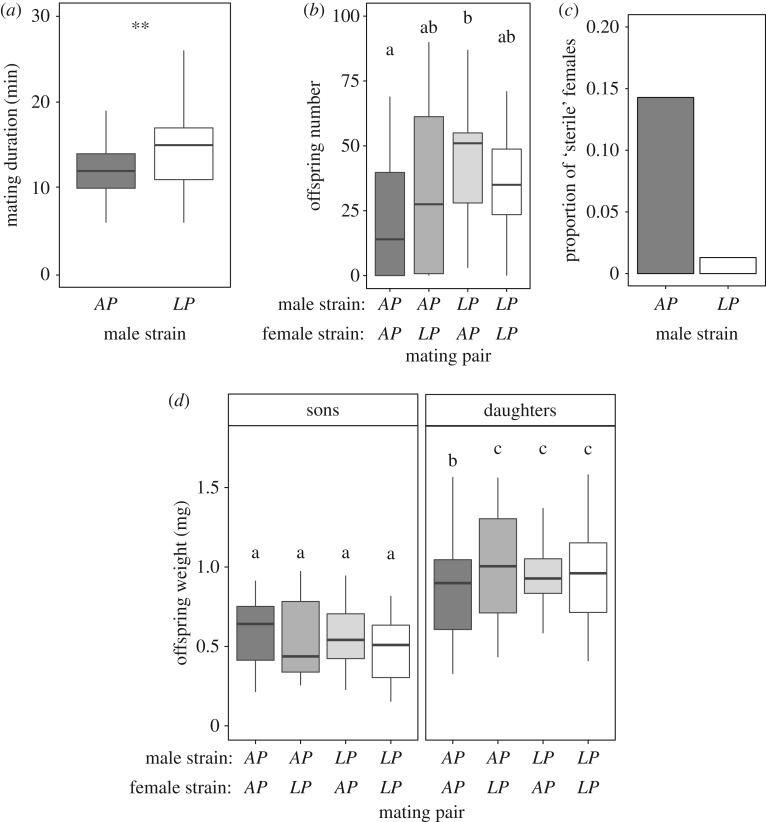
Direct and trans-generational effects of male and female gut microbiota. (*a*) Mating duration (minutes). **p < 0.01. (*b*) Short-term reproductive success (offspring number) of mating pairs. (*c*) Proportion of females with zero offspring production after mating (i.e. ‘sterile’). (*d*) Sons' and daughters' body mass (in milligrams). SNK *post hoc* test (*α* = 0.05).

Germ-free flies of both sexes were re-inoculated with either *AP* or *LP*. We then mated males and females of all treatments in combinations and measured latency of virgin females to mate as a proxy for male attractiveness, mating duration as a proxy for male mating investment, short-term (72 h) offspring production as a proxy for reproductive success [13] as well as offspring body mass as a proxy for offspring quality (e.g. [14], figure 1). We then tested whether homotypic and heterotypic mating could affect individuals' reproductive success, whether this effect was a result of the interaction between the gut microbiota of both sexes or if there was a sex-specific effect of gut microbiota (figure 1; see the electronic supplementary material). Male strain but not female strain of gut bacteria had a significant effect on the duration of mating (Male strain: *F*_1,72_ = 8.533, *p* = 0.004), with mating pairs with *LP-*infected males mating relatively longer (figure 2*a*). This effect was also seen in offspring production of the mating pair, where pairs with *LP-*infected males had higher offspring production (Male strain: *F*_1,72_ = 5.152, *p* = 0.026) (figure 2*b*). There was also a borderline non-significant interaction Male strain × Female strain on offspring production (Male strain × Female strain: *F*_1,71_ = 3.813, *p* = 0.054), where mating pairs with *LP*-infected males and *AP*­-infected females had higher short-term offspring production (figure 2*b*; see the electronic supplementary material for full analysis). The effect of male gut bacteria strain on offspring production of the mating pair was driven by a higher proportion of females failing to produce any offspring after mating (i.e. zero offspring production) (Male strain: *F*_1,75_ = 12.641, *p* < 0.001), whereby females mating with *AP*-infected males were more likely to have zero offspring production than females mating with *LP*-infected males (figure 2*c*). When females that failed to produce offspring were removed from the analysis, there was no effect of either male strain (*F*_1,62_ = 0.413, *p* = 0.522), female strain (*F*_1,61_ = 0.404, *p* = 0.527) or their interaction (*F*_1,58_ = 0.293, *p* = 0.092). Together, our results show no direct evidence for advantages of homotypic mating as suggested previously [15] but suggest that the strain of male gut bacterium is important for mating investment and reproductive success of the mating pair.

We then asked whether homotypic and heterotypic mating affected the next generation or if there was also a sex-specific effect of gut microbiota on trans-generational effects of the gut bacteria. We found an interaction of Male strain × Female strain on daughters', but not sons', body mass (see the electronic supplementary material, daughters: *F*_1,295.7_ = 5.487, *p* = 0.020; sons: *F*_1,300.6_ = 0.026, *p* = 0.873), showing that daughters' body mass can be significantly reduced when both the father and the mother are re-inoculated with *AP* strain compared with the other pairings (figure 2*d*). This result partly contradicts our prediction that homotypic mating confers indirect fitness advantages to the mating pair through offspring quality. Body mass in *D. melanogaster* is positively associated with fecundity [16,17], courtship activity [18], fertilization success [17] and attractiveness [16,19], which together can modulate the strength of sexual selection in the population [17]. Thus, the trans-generational effects due to parental gut bacteria found in our study can potentially have long-lasting implications for the evolution of a population. Future studies should address the impact of gut microbiota on the operation of sexual selection, and also investigate the implications of our findings for wild populations given that the distribution and abundance of *AP* and *LP* in wild flies differ markedly from laboratory-adapted strains [11].

Our results show the overall negative effects of *AP* re-inoculation on mating investment, offspring production, and offspring body mass of mating pairs relative to *LP* re-inoculation. The mechanisms underlying these effects remain unknown. We speculate that *AP* infection could modulate the immunity–reproduction trade-off in *D. melanogaster* because *AP* induces the expression of Pvf2, a key ligand that increases haemocyte circulation [20], in an NF-κB-dependent manner, which helps the organism to rapidly assemble a response when facing viral immune challenges [21]. However, in the absence of immune challenges, such as in the controlled lab environment, the sustained *AP-*induced Pvf2 production could maintain an immune response that compromises reproduction [22]. Whether this, or another yet unknown pathway, is responsible for the negative reproductive effects of *AP* re-inoculation remains a key area for future investigations. Overall, more studies are needed to better understand the molecular mechanisms underlying the interactions between *D. melanogaster* and its commensal bacteria.

## 3. Conclusion

In this study, we revealed novel direct and trans-generational effects of the gut bacteria on mating and reproductive behaviour of *D. melanogaster*. Results show a positive effect of *LP* strain on reproductive success when present in males relative to males re-inoculated with *AP,* and a negative effect of *AP* strain on offspring weight when present in both parents relative to *LP-*inoculated parents. While it has been previously shown that gut bacteria can modulate social recognition and mate choice, this study shows for the first time that the composition of the gut bacteria contributes to reproductive success in *D. melanogaster*. Our results are likely to be widespread given the far-reaching effects of the gut microbiota in a range of taxa [23,24], and show the potential implications of the gut bacteria in key evolutionary processes such as sexual selection.

## 4. Material and methods

### (a) Fly stock

We used wild-type OregonR strain. All experiments were performed at 25°C, 65% humidity and 13 h L : 11 h D cycle (see the electronic supplementary material for details).

### (b) Egg collection and gnotobiotic flies

We controlled for any potential effect of larval density on body size of our flies through Clancy & Kennington [25]'s protocol, and also controlled for familiarity by pipetting eggs into more than 70 vials and pooling all adults before sampling experimental individuals which reduce the likelihood of pairing familiar individuals for the mating trials. We dechorionated eggs as in [26] (see the electronic supplementary material for details). We pipetted the eggs at a density of approximately 1 larva ml^−1^ of food in vials with approximately 5 ml of food with antibiotics (50 µg ml^−1^ streptomycin and 25 µg ml^−1^ kanamycin, final concentration in the diet).

### (c) Bacterial strains and re-inoculation

*LP* and *AP* were isolated from our stocks, and were identified through Sanger 16S sequencing (AGRF^®^ Australia) and morphology of colonies (see the electronic supplementary material for sequences and details of sequencing). Adult flies were re-inoculated in vials with MRS agar inoculated with 1 ml of bacteria cultures at a concentration of 10^8^ CFU ml^−1^, and transferred to fresh infection vials every 48 h for four consecutive days (see the electronic supplementary material for details). We checked the efficacy of our protocol throughout our experiments through MRS agar culture plates. Our protocol successfully enriched the flies' gut bacteria in the direction expected (sex: CFU per fly ± s.d., males *AP*-infected = 4.6 × 10^3^ ± 4.2 × 10^3^; males *AP*-conventional = 2.93 × 10^4^ ± 1.57 × 10^4^; females *AP*-infected = 4.93 × 10^3^ ± 3.97 × 10^3^; females *AP*-conventional = 7.03 × 10^4^ ± 2.81 × 10^4^; males *LP*-infected = 2.26 × 10^4^ ± 1.85 × 10^4^; males *LP*-conventional = 9.20 × 10^4^ ± 2.80 × 10^4^; females *LP*-infected = 2.22 × 10^4^ ± 1.77 × 10^4^; females *LP*-conventional = 9.21 × 10^4^ ± 4.01 × 10^4^)).

### (d) Data analysis

We had 17 replicate mating pairs per treatment in a fully factorial design (*N* = 68). Each pair was allowed to interact and mate for 4 h when we measured latency of virgin females to mate and mating duration. After this period, females were allowed to oviposit for approximately 72 h before being discarded. Adult offspring was counted 13 days after oviposition (i.e. ‘short-term reproductive success'). We sampled randomly the pool of offspring per replicate per treatment for body mass, assessed in a fine-scale Sartorium balance with precision of 0.001 g and performed analyses using linear mixed models (see the electronic supplementary material; *N: AP×AP* = 154, *AP×LP* = 157, *LP×AP* = 152 and *LP×LP* = 142). We used general linear models for the analyses of female latency to mate and mating duration, and generalized linear models for short-term reproductive success and proportion of virgin females that failed to produce offspring after mating (see the electronic supplementary material). All plots are of the raw data and all analyses were performed in R [27] with the exception of the offspring body mass analyses, which were performed in SPSS [28].

## Supplementary Material

Methods detail, full analyses output and 16S DNA sequences

## Data accessibility

The data are available in Dryad (http://dx.doi.org/10.5061/dryad.5kv17) [29].
