# Supplementary material for: Direct and trans-generational effects of male and female gut microbiota in Drosophila melanogaster
Source: Biol Lett. 2017 Jul 19;13(7):20160966. doi: 10.1098/rsbl.2016.0966 (PMC5543016; doi:10.1098/rsbl.2016.0966)
Supplement: Methods detail, full analyses output and 16S DNA sequences [file rsbl20160966supp1.pdf]

Supplementary Information: "Direct and trans-generational effects of male and female gut microbiota in *Drosophila melanogaster*"

Authors: Juliano Morimoto[1,2,*], Stephen J Simpson[1,3], Fleur Ponton[4]

Affiliations:

1 - Charles Perkins Centre, University of Sydney, Camperdown NSW 2006, Australia

2 - Programa de Pós-Graduação em Ecologia e Conservação, Federal University of Paraná, Curitiba, Brazil, 19031, CEP: 81531-990

3- School of Biological Sciences, University of Sydney, Sydney 2006, Australia

4 - Department of Biological Sciences, Macquarie University, North Ryde, NSW 2109, Australia

*To whom correspondence should be addressed.
E-mail: juliano.morimoto@sydney.edu.au

Keywords: sexual conflict, mate choice, holobiont, polyandry, microbiome

Supplementary Methods

Fly stock

We used inbred wild-type OregonR strain from Bloomington maintained in large populations (>1,000 individuals) with overlapping generations. All stocks were maintained and all experiments performed at 25ºC with humidity 65% in a light:dark cycle of 13:11 and in standard Bloomington yeast-maize-molasses diet with excess live yeast granules.

Egg dechorionation and gut bacteria re-inoculation

We dechorionated eggs as follows: four washes in a solution of 0.6% bleach followed by three washes in 0.1M PBS buffer pH 6.8 (see Koyle et al. 2016). For re-inoculation, recently emerged (~6h) gnotobiotic flies were collected on ice anaesthesia, sexed and transferred to same-sex infection vials containing 15-20 individuals before infection. Adult flies were then re-inoculated in vials with MRS agar inoculated with 1mL of bacteria cultures at concentration of 10^8 CFU/mL, and were transferred to fresh infection vials every 48h for 4 consecutive days.

Mating trials and statistical analyses

We placed a male and a female (see Figure 1 for details) in vials (Drosophila vial: 25 X 95 mm) with 4mL of standard autoclaved yeast-maize-molasses diet and allowed the pair to interact and mate for 4h. We fitted a linear mixed model for sons and daughters separately, including replicate as a random variable while the interaction of male strain and female strain as fixed effects. For the latency of females to mate, mating duration and offspring body mass we used general linear models and ANOVA with male and female strain as fixed effects (see ESM for details). We BoxCox transformed latency to mate or remate (i.e. latency^0.1) to fit the normality assumption. For mating duration, we used a similar model but which included a covariate to control for female latency to mate. For short-term reproductive success, we fitted a quasipoisson Generalized Linear Model (GLM) to account for overdispersion of the data while controlling for the latency of females to mate and mating duration. For the proportion of virgin females that fail to produce offspring after mating we fitted a quasibinomial GLM that also account for the dispersion of the data. P-values were obtained from F-statistics.

Supplementary Results

Table S1 - Full ANOVA analysis of the effects of male and female strain on mating pairs' reproduction. Bold – $p < 0.05$.

Factors	Response							
	Latency to mate		Mating duration		Offspring production		Percentage of sterile pairs	
	F-value	*p-value*	*F-value*	*p-value*	*F-value*	*p-value*	*F-value*	*p-value*
**Male strain**	0.421	0.518	8.533	**0.004**	5.152	**0.026**	12.641	**<0.001**
**Female strain**	0.573	0.451	0.456	0.516	0.145	0.703	0.000	0.976
**Latency to mate**	-	-	7.773	**0.006**	0.900	0.345	-	-
**Mating duration**	-	-	-	-	16.614	**<0.001**	-	-
**Male strain*Female strain**	0.001	0.969	0.036	0.848	3.813	0.054	1.677	0.199

Table S2 - Full ANOVA analysis of the effects sex-specific transgenerational effects of male and female strain on offspring weight. Bold – $p < 0.05$. Replicate was included as a random variable to control for pseudoreplication.

Factors	Offspring body mass			
	Daughters		Sons	
	F-value	*p-value*	*F-value*	*p-value*
**Male strain**	**7.400**	**0.007**	1.715	0.187
**Female strain**	0.093	0.761	1.485	0.224
**Male strain*Female strain**	**5.487**	**0.020**	0.026	0.873

Bacterial DNA sequencing and strain identification

The AGRF 16S Bacterial Sequencing employs universal primers to interrogate an approximate 800bp region of the 16S ribosomal RNA (see Table S3). To identify the strains, the sequences were blasted against AGRF's in-house 16S database using Paracel Blast. ARGF® database is manually collated from the Greengenes database of 16S rRNA genes from the Lawrence Berkeley National Laboratory [1]. First hits of the blast confirmed the identity of our sequences as *Lactobacillus plantarum* and *Acetobacter pomorum*. To corroborate this identification, we also ran BLAST against the NCBI microbial database with the following settings: Database>All genomes; Organism> taxid: 1578 [for *L. plantarum* search], and taxid: 434 [for *A. pomorum* search]; Optimize for: "Highly similar sequences (megablast)". The results are given in Table S4.

Table S3 – *Acetobacter pomorum* and *Lactobacillus plantarum* 16S gene DNA partial sequences

Acetobacter pomorum (657bp)
ACATGCAAGTCGCACGAAGGTTTCGGCCTTAGTGGCGGACGGGTGAGTAACG<br>CGTAGGTATCTATCCATGGGTGGGGGATAACACTGGGAAACTGGTGCTAATA<br>CCGCATGACACCTGAGGGTCAAAGGCGTAAGTCGCCTGTGGAGGAGCCTGCG<br>TTTGATTAGCTAGTTGGTGGGGTAAAGGCCTACCAAGGCGATGATCAATAGCT<br>GGTTTGAGAGGATGATCAGCCACACTGGGACTGAGACACGGCCCAGACTCCT<br>ACGGGAGGCAGCAGTGGGGAATATTGGACAATGGGGGCAACCCTGATCCAGC<br>AATGCCGCGTGTGTGAAGAAGGTCTTCGGATTGTAAAGCACTTTCGACGGGG<br>ACGATGATGACGGTACCCGTAGAAGAAGCCCCGGCTAACTTCGTGCCAGCAG<br>CCGCGGTAATACGAAGGGGGCTAGCGTTGCTCGGAATGACTGGGCGTAAAGG<br>GCGTGTAGGCGGTTTGTACAGTCAGATGTGAAATCCCCGGGCTTAACCTGGG<br>AGCTGCATTTGATACGTGCAGACTAGAGTGTGAGAGAGGGTTGTGGAATTCC<br>CAGTGTAGAGGTGAAATTCGTAGATATTGGGAAGAACACCGGTGGCGAAGGC<br>GGCAACCTGGCTCATTACTGACGCTGAGGCGC
Lactobacillus plantarum (733bp)
ACATGCAAGTCGAACGAACTCTGGTATTGATTGGTGCTTGCATCATGATTTACA<br>TTTGAGTGAGTGGCGAACTGGTGAGTAACACGTGGGAAACCTGCCCAGAAGC<br>GGGGGATAACACCTGGAAACAGATGCTAATACCGCATAACAACTTGGACCGC<br>ATGGTCCGAGTTTGAAAGATGGCTTCGGCTATCACTTTTGGATGGTCCCGCGGC<br>GTATTAGCTAGATGGTGGGGTAACGGCTCACCATGGCAATGATACGTAGCCGA<br>CCTGAGAGGGTAATCGGCCACATTGGGACTGAGACACGGCCCAAACTCCTACG<br>GGAGGCAGCAGTAGGGAATCTTCCACAATGGACGAAAGTCTGATGGAGCAAC<br>GCCGCGTGAGTGAAGAAGGGTTTCGGCTCGTAAAACTCTGTTGTTAAAGAAGA<br>ACATATCTGAGAGTAACTGTTCAGGTATTGACGGTATTTAACCAGAAAGCCAC<br>GGCTAACTACGTGCCAGCAGCCGCGGTAATACGTAGGTGGCAAGCGTTGTCCG<br>GATTTATTGGGCGTAAAGCGAGCGCAGGCGGTTTTTTAAGTCTGATGTGAAAG<br>CCTTCGGCTCAACCGAAGAAGTGCATCGGAAACTGGGAAACTTGAGTGCAGA<br>AGAGGACAGTGGAACTCCATGTGTAGCGGTGAAATGCGTAGATATATGGAAG<br>AACACCAGTGGCGAAGGCGGCTGTCTGGTCTGTAACTGACGCTGAGG

Table S4 – Strain identification. BLAST results for the identification of *Acetobacter pomorum* and *Lactobacillus plantarum* strains.

***Acetobacter pomorum* identification**					
Hit	***Description***	***Max score***	***Total score***	***Identity***	***Sequence ID***
1	*Acetobacter pomorum strain DmCS …*	1214	1214	100%	JOKL01000050.1
2	*Acetobacter pomorum DM001 …*	1214	1214	100%	AEUP01000040.1
3	*Acetobacter pasteurianus strain SRCM100623 …*	1208	1208	99%	LYUD01000173.1
***Lactobacillus plantarum* identification**					
Hit	***Description***	***Max score***	***Total score***	***Identity***	***Sequence ID***
1	*Lactobacillus plantarum* strain CAUH2, complete genome	1354	7257	100%	NZ_CP015126.1
2	*Lactobacillus plantarum* isolate 1 genome assembly …	1347	1347	99%	FGVL01000051.1
3	*Lactobacillus plantarum* strain NAB1NODE_6 …	1349	1349	99%	LUXN01000006.1

Offspring density and body weight

We found no evidence for a negative correlation between the average number of offspring and the average body mass of the offspring (t-value = -0.662, p = 0.509).

Supplementary References

[1] DeSantis, T.Z., Hugenholtz, P., Larsen, N., Rojas, M., Brodie, E.L., Keller, K., Huber, T., Dalevi, D., Hu, P. & Andersen, G.L. 2006 Greengenes, a chimera-checked 16S rRNA gene database and workbench compatible with ARB. *Appl. Environ. Microbiol.* **72**, 5069-5072.
